# Vitrectomy with macular peeling in eyes with vitreomacular interface disorders and nonexudative age-related macular degeneration

**DOI:** 10.1007/s00417-025-06848-z

**Published:** 2025-05-01

**Authors:** Marco Pellegrini, Ginevra Giovanna Adamo, Chiara Vivarelli, Laura Sarti, Pietro Maria Talli, Francesco Nasini, Francesco Parmeggiani, Marco Mura

**Affiliations:** 1 Department of Translational Medicine, University of Ferrara, Ferrara, Italy; 2 Sant’Anna University Hospital, Via A. Moro 8, 44124 Cora, Ferrara, Italy; 3 Department of Ophthalmology, Ospedali Privati Forlì “Villa Igea”, Forlì, Italy; 4 King Khaled Eye Specialist Hospital, Riyadh, Saudi Arabia

**Keywords:** Age-related macular degeneration, Vitrectomy, Vitreoretinal-surgery, Vitreoretinal interface disorders

## Abstract

**Background:**

The aim of the study was to assess the outcomes of pars plana vitrectomy and macular peeling for vitreomacular interface disorders in eyes with coexisting nonexudative age-related macular degeneration (AMD), and to compare the rate of AMD progression in operated and fellow eyes.

**Methods:**

This retrospective comparative study included patients with bilateral nonexudative AMD and unilateral vitreomacular interface disorder who underwent pars plana vitrectomy with internal limiting membrane peeling. Controlateral unoperated eyes were used as a control group. Cox proportional hazards regression analysis was used to compare the incidence of macular neovascularization, geographic atrophy and progression to late AMD in operated eyes and fellow eyes.

**Results:**

142 eyes of 71 patients were included. The mean follow-up duration was 17.5 ± 15.5 months. Best corrected visual acuity significantly improved in operated eyes (from 0.47 ± 0.25 to 0.21 ± 0.18 logMAR; *p* < 0.001), while no significant difference was observed in fellow eyes (from 0.18 ± 0.22 to 0.33 ± 0.56 logMAR; *p* = 0.055). Central retinal thickness improved in operated eyes (from 0402.9 ± 79.9 to 296.1 ± 48.4 µm; *p* < 0.001), while no change in fellow eyes was observed (from 296.1 ± 48.4 to 312.7 ± 110.6 µm; *p* = 0.205). Vitrectomy was not associated with the risk of developing macular neovascularization (hazard ratio [HR] = 0.30; 95% confidence intervals (CI) = 0.08–1.09; *p* = 0.068); geographic atrophy (HR = 1.01, 95% CI = 0.32–3.12; *p* = 0.990) nor progression to late AMD (HR = 0.64, 95% CI = 0.28–1.49; *p* = 0.307).

**Conclusions:**

Pars plana vitrectomy with macular peeling for vitreomacular interface disorders in eyes with coexisting nonexudative AMD is associated with positive visual and functional outcomes, with no shot-term increased risk of AMD progression.

## Key messages

***What is known***

• Some surgeons are reluctant to perform vitreoretinal surgery in eyes with AMD due to the concern that it may accelerate its progression.

***What is new***

• Virectomy with macular peeling in eyes with coexisting nonexudative AMD is associated with positive visual and functional outcomes.

• Vitreoreoretinal surgery does not seem to confer any additional risk of AMD progression

## Introduction

Age-related macular degeneration (AMD) and disorders of the vitreoretinal interface including epiretinal membranes and macular holes are common causes of visual impairments in older individuals [1–3]. Pars plana vitrectomy remains the gold-standard treatment for improving vision in eyes with vitreoretinal interface diseases. Although it remains uncertain whether vitreoretinal surgery may affect the risk of AMD, some retinal surgeons remain reluctant to perform vitrectomy in eyes with AMD due to the concern that the mechanical trauma to the macula, breakdown of the blood-ocular barrier and/or endoillumination retinal phototoxicity may accelerate AMD progression [4, 5]. On the other hand, abnormal vitreomacular adhesion and traction may represent a risk factor for the progression to neovascular AMD and antagonize the effect of anti-neovascular growth factor treatment [6, 7]. Moreover, it has been demonstrated that delaying vitrectomy in eyes with vitreomacular interface disorders can negatively affect visual and anatomical outcomes [8, 9].

The aim of this study was to report the clinical outcomes of vitrectomy with macular peeling in eyes with non-exudative AMD and concomitant vitreomacular interface disorders. To assess the possible association between vitrectomy and AMD progression, the rate of progression in the operated eye and fellow eye were compared.

## Methods

This retrospective comparative study included patients with bilateral non-exudative AMD and unilateral vitreomacular interface disorder who underwent vitrectomy with macular peeling at the Sant’Anna University Hospital (University of Ferrara, Ferrara, Italy) between January 2019 and September 2023. Non-vitrectomized fellow eyes were used as a control group. Inclusion criteria were: bilateral AREDS category 3 AMD (at least one large [≥ 125 μm] drusen or extensive intermediate [≥ 63 μm] drusen); pars plana vitrectomy with internal limiting membrane (ILM) peeling for unilateral vitreomacular interface disorder. Exclusion criteria were: geographic atrophy, macular neovascularization or disciform scarring in either eye at baseline; other retinal diseases such as retinal detachment, diabetic retinopathy, retinal vascular occlusion in either eye; history of vitrectomy or vitreomacular interface disorder in the fellow eye; follow-up of less than 6 months; lack or poor quality of raster optical coherence tomography (OCT) scans at any follow-up visit. The study adhered to the tenets of the 2013 Declaration of Helsinki and was prospectively approved by the local Institutional Review Board.

Patients were seen before surgery and after surgery at month 1, 3, 6, 12 and yearly thereafter. At each follow-up examination, all patients underwent a complete ophthalmologic examination including best-corrected visual acuity (BCVA), applanation tonometry, slit-lamp biomicroscopy, fundoscopy, color fundus photograph (Clarus 500; Carl Zeiss Meditech Inc., Dublin, USA) and macular OCT (Spectralis; Heidelberg Engineering, Carlsbad, Germany). Fluorescein angiography, fundus autofluorescence (Spectralis; Heidelberg Engineering, Carlsbad, Germany) and/or OCT angiography (Cirrus 6000; Carl Zeiss Meditech Inc., Dublin, USA) were also performed in selected cases to ascertain the presence of macular neovascularization or geographic atrophy. Retinal imaging was retrospectively reviewed by two independent retinal specialists to confirm the diagnoses. Macular neovascularization was diagnosed based on the results of multimodal imaging including macular OCT, fluorescein angiography and OCT angiography. Geographic atrophy was diagnosed in presence of complete retinal pigment epithelial and outer retinal atrophy (cRORA), defined as a region of hypertransmission and attenuation/disruption of the RPE of at least 250 μm in diameter with overlying photoreceptor degeneration on macular OCT [10]. The diagnosis was confirmed with fundus autofluorescence. Central retinal thickness (CRT) was manually measured in each OCT scan using the built-in software caliper.

All patients underwent 25-gauge pars plana vitrectomy (Constellation Vision System; Alcon Laboratories Inc., Fort Worth, TX, USA) using a wide-angle noncontact viewing system (Resight; Carl Zeiss Meditec AG, Jena, Germany). In all phakic patients, combined phacoemulsification, intraocular lens implantation and pars plana vitrectomy was performed. After staining of the vitreous with triamcinolone acetonide, a complete posterior vitreous detachment was induced, if not already present. The epiretinal membrane (ERM)/ILM complex was stained with MembraneBlue Dual, (TrypanBlue 0.15% + Brilliant Blue G 0.025%, DORC, Zuidland, The Netherlands) and peeled using end-gripping vitreoretinal forceps. Gas tamponade was performed with 20% sulfur hexafluoride gas in eyes with full-thickness macular holes, and with air in all remaining cases.

Statistical analysis was performed using R (version 4.0.0) and RStudio (version 1.2.5042) software. Cox proportional hazards regression analysis was used to compare the incidence of macular neovascularization, geographic atrophy and late AMD (defined by the presence of macular neovascularization, geographic atrophy, serous epithelial detachment or disciform scarring) between operated and fellow eyes. The Wilcoxon signed-rank test was used to compare continuous variables between groups. A *p*-value of less than 0.05 was considered statistically significant.

## Results

A total of 142 eyes of 71 patients were included in the study (71 eyes as study group and 71 eyes as control group). Baseline demographical and clinical variables are reported in Table 1. The primary indication for surgery was epiretinal membrane in 73.2% of eyes, full-thickness macular hole in 15.5%, lamellar macular hole in 8.5% and vitreomacular traction syndrome in 2.8%. Twenty-eight eyes (39.4%) were pseudophakic at the time of vitrectomy, the remaining 43 phakic eyes (60.6%) underwent combined cataract extraction and pars plana vitrectomy. The mean follow-up duration after surgery was 17.5 ± 15.5 months (range: 6–67 months).

**Table 1:** Baseline characteristics of included patients

Characteristic	Value
Women, n (%)	40 (56.3)
Age, mean (± SD)	76.7 ± 6.1
Pseudophakic, n (%)	28 (39.4)
Indication for surgery	
ERM, n (%)	52 (73.2)
FTMH, n (%)	11 (15.5)
LMH, n (%)	6 (8.5)
VMT, n (%)	2 (2.8)

*SD*; standard deviation, *ERM*; epiretinal membrane, *FTMH*; full-thickness macular hole, *LMH*; lamellar macular hole, *VMT*; vitreo-macular traction

Table 2 shows the functional and anatomical outcomes after surgery. Preoperatively, mean BCVA was significantly lower in study eyes compared to control eyes (*p* < 0.001). At the last follow-up, mean BCVA significantly improved in study eyes (*p* < 0.001), while no significant difference in fellow eyes was observed (*p* = 0.055). Study eyes showed a significantly higher CRT than fellow eyes before surgery. After surgery, CRT significantly decreased in study eyes (*p* < 0.001), while no significant change was observed in fellow eyes was (*p* = 0.205). Full-thickness macular holes closed in 100% of eyes.

**Table 2:** Functional and anatomical changes following vitrectomy with internal limiting membrane peeling in operated eyes and fellow eyes

Parameter	Operated eyes	Fellow eyes	*P*-value
BCVA, (logMAR)			
Before surgery	0.47 ± 0.25	0.18 ± 0.22	< 0.001
After surgery	0.21 ± 0.18	0.33 ± 0.56	0.049
CRT (µm)			
Before surgery	402.9 ± 79.9	296.1 ± 48.4	< 0.001
After surgery	351.2 ± 68.7	312.7 ± 110.6	0.023

*BCVA*; Best-corrected visual acuity, *CRT*; Central retinal thickness

**Fig. 1:**
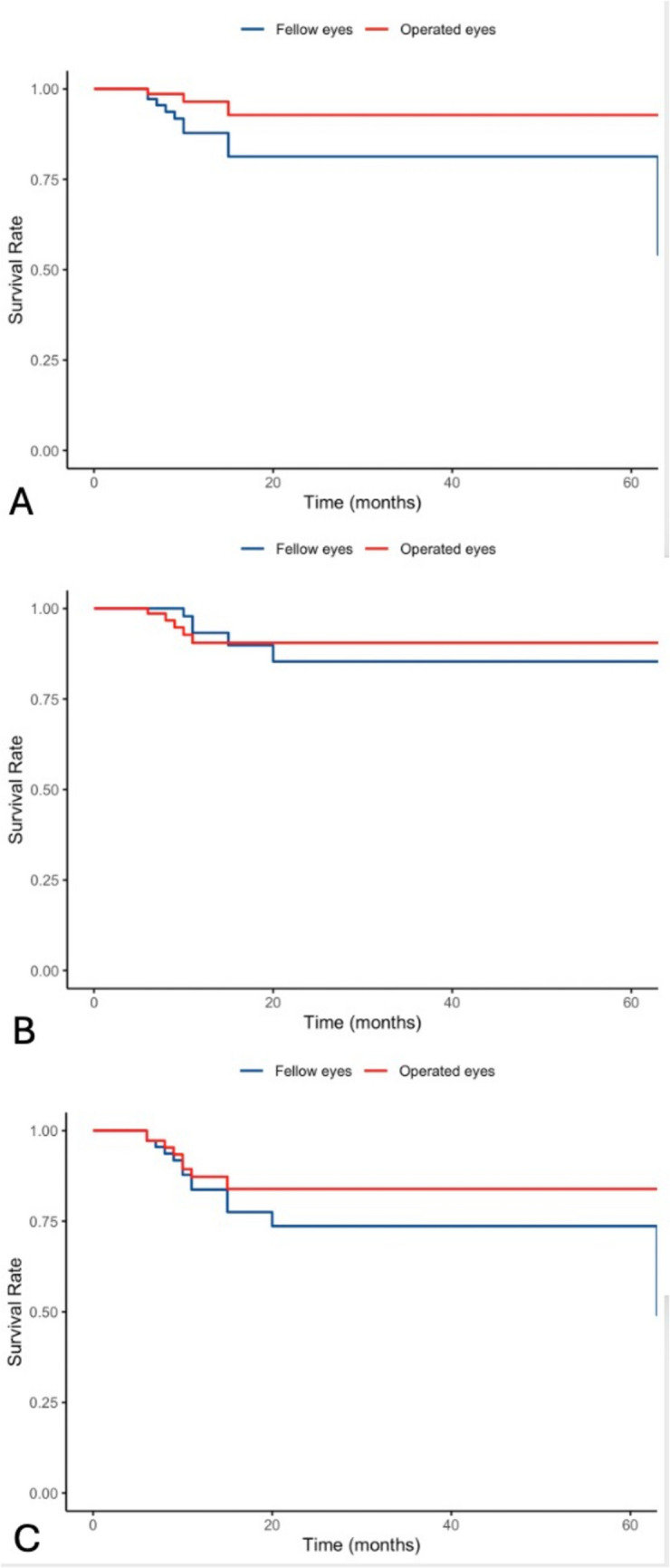
Kaplan–Meier curve of macular neovascularization (part **A**), geographic atrophy (part **B**) and progression to late AMD (part **C**) following vitrectomy with macular peeling.

**Fig. 2:**
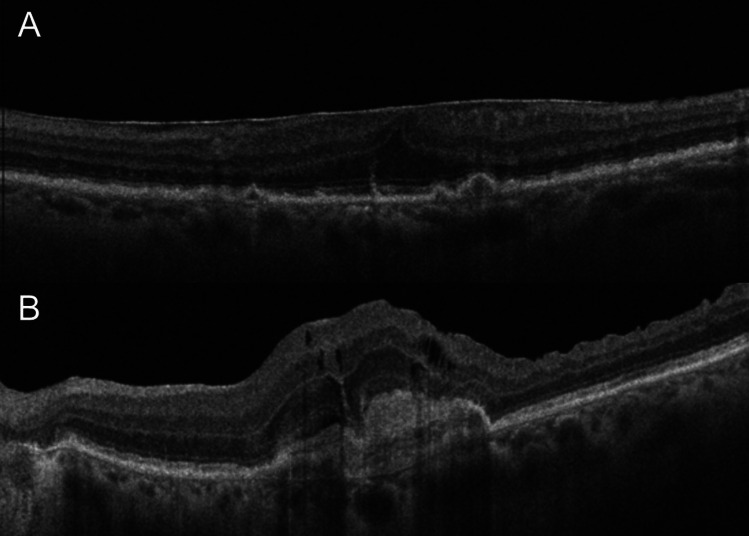
Structural optical coherence tomography in a representative patient who underwent vitrectomy and epiretinal membrane peeling. **A**, before surgery, soft drusen and an epiretinal membrane are visible. **B**, After surgery, the patient developed macular neovascularization

A total of 3 operated eyes (4.2%) and 10 control fellow eyes (14.1%) developed macular neovascularization (Fig. 2). Table 3 shows the results of the Cox regression analysis, which showed no effect of surgery on the risk of developing macular neovascularization (*p* = 0.068). Geographic atrophy (cRORA) developed in 6 operated eyes (8.5%) and 6 fellow eyes (8.5%). No association between surgery and the development of geographic atrophy was observed (*p* = 0.990). In total, 9 operated eyes (12.7%) and 14 control eyes (19.7%) progressed to late AMD during the follow-up. No association between surgery and the progression to late AMD was observed (*p* = 0.307) (Fig. 1).

**Table 3:** Univariate Cox regression analysis on the association between vitrectomy with internal limiting membrane peeling and the risk of developing MNV, GA and late AMD

Outcome	HR	95% CI	P-value
MNV	0.30	0.08–1.09	0.068
GA	1.01	0.32–3.12	0.990
Late AMD	0.64	0.28–1.49	0.307

*MNV*; Macular neovascularization, *GA*; Geographic atrophy, *AMD*; Age-related macular degeneration, *HR*; Hazard ratio, *CI;* Confidence interval

## Discussion

Previous studies suggested a possible relationship between vitreomacular interface disorders and AMD [11–14]. Although some authors hypothesized a possible pathogenic role of vitreomacular traction in the development of neovascular AMD [6, 11, 15], others postulated that, rather than a causative factor, abnormal vitreomacular adhesion may represent a consequence of the chronic inflammation and exudation occurring in eyes with macular neovascularization [12, 13]. Therefore, the possible role of vitreoretinal surgery in the prevention or treatment of neovascular AMD is still unclear (Fig. 2).

The present study demonstrated good functional and visual outcomes following pars plana vitrectomy and macular peeling in eyes with vitreomacular interface disorders and concomitant non-exudative AMD. In particular, after a mean follow-up of 17.5 months, an improvement of 0.26 logMAR in BCVA and a significant decrease of CRT were observed. This result is in line with those from previous reporting mean improvements in logMAR BCVA ranging from 0.14 to 0.19 in eyes with non-exudative AMD undergoing epiretinal membrane surgery [16–18].

The main concern of performing vitreoretinal surgery in eyes with is that the mechanical trauma to the macula may accelerate the progression of the disease. However, the available evidence regarding the association between vitreoretinal surgery and AMD is still inconclusive. While Roller et al. suggested that vitrectomy could reduce the risk of progression to macular neovascularization or geographic atrophy, [4] Muftuoglu et al. did not identify any effect of surgery of surgery on AMD progression and drusen volume. [5] On the contrary, two recent retrospective studies from Obeid et al. and Furashova et al. reported higher rates of progression to neovascular AMD in vitrectomized eyes compared to fellow eyes. [17, 18] It should be noted that all the above-mentioned studies have relatively small sample sizes ranging from 22 to 42 patients.

To our knowledge, this represents the largest series evaluating possible association between vitrectomy and AMD progression. The “paired eye” survival analysis revealed no association between surgery and the risk of macular neovascularization, cRORA nor progression to late AMD. On the contrary, the rate of macular neovascularization was lower in vitrectomized eyes (4.2%) than in fellow eyes (14.2%). Possible explanations for this finding might include the elimination of tractional forces after the surgical induction of posterior vitreous detachment [4, 18], as well as the increased oxygen tension in the vitreous with improved oxygen diffusion to the outer retina [19, 20]. However, since the reduced risk of macular neovascularization in vitrectomized eyes did not reach statistical significance, this result should be interpreted with great caution.

Since anti-VEGF agents remain the mainstay treatment for neovascular AMD, the possible effect of vitreoretinal surgery on the outcomes of anti-VEGF therapy deserves attention. It has been previously demonstrated that eyes with vitreomacular traction or epiretinal membranes have decreased response to anti-VEGF agents, requiring more intensive treatment regimens [20–22]. On the other hand, animal studies suggested that the intraocular clearance of anti-VEGF agents in vitrectomized eyes may be increased, thus reducing treatment efficacy [23, 24]. Therefore, the possible effect of vitrectomy on the response to anti-VEGF agents remains controversial.

The paired-eye design employed in this study has some important advantages, including the natural matching of baseline values between groups, the implicit control for potential genetic and environmental confounders and the increased statistic power. However, there are also some important limitations to the current study, such as its retrospective design and the inconsistent follow-up duration in the study population. Moreover, cases of combined vitrectomy and cataract surgery were included, and this might have affected the rates of AMD progression. Finally, the median follow-up of the study was relatively short, while AMD can slowly progress over the course of several years. Therefore, longer longitudinal studies are still required to evaluate the long-term effect of vitreoretinal surgery on AMD progression.

In conclusion, the current study demonstrated positive functional and anatomic results of pars plana vitrectomy with macular peeling in eyes with vitreomacular interface disorders and concomitant AMD. Vitrectomy does not seem to confer an additional risk of AMD progression on the short-term. Further prospective studies are required to establish whether performing vitreoretinal surgery in selected cases may decrease the risk of macular neovascularization.
